# Comprehensive analysis of expression signature and immune microenvironment signature of biomarker Endothelin Receptor Type A in stomach adenocarcinoma

**DOI:** 10.7150/jca.68673

**Published:** 2022-03-28

**Authors:** Zhengguang Wang, Kangchun Wang, Xue Yu, Moye Chen, Yaqi Du

**Affiliations:** 1 Department of Gastroenterology, The First Affiliated Hospital of China Medical University, Shenyang, Liaoning, China.; 2 Department of Orthopedics, The First Affiliated Hospital of China Medical University, Shenyang, Liaoning, China.; 3 Department of Organ transplantation and Hepatobiliary, The First Affiliated Hospital of China Medical University, Shenyang, Liaoning, China.; 4 Department of Paediatrics, Wuhan Children's Hospital (Wuhan Maternal and Child Healthcare Hospital), Tongji Medical College, Huazhong University of Science and Technology, Wuhan, China.

**Keywords:** EDNRA, Tumor biomarker, Immune checkpoint, M2 macrophage, Immunoregulatory

## Abstract

**Background:** EDNRA (Endothelin Receptor Type A) is closely associated with tumor progression in many tumor types. However, the functional mechanism of EDNRA in stomach adenocarcinoma (STAD) remains to be elucidated.

**Methods:** ENDRA expression levels in STAD were assessed. A Receiver Operating Characteristic (ROC) curve was constructed to measure the diagnostic value of EDNRA. The correlation between ENDRA expression levels and patient clinical-pathological characteristics was analyzed. The survival and prognostic significance were validated using Kaplan-Meier and Cox regression and confirmed by the immunohistochemistry cohorts. Differentially expressed genes of EDNRA in STAD were determined, and EDNRA related functional enrichment and biological pathways involved in STAD were obtained by Gene-Set Enrichment Analysis (GSEA). The correlation between EDNRA expression in STAD and immune cell infiltration was assessed using the CIBERSORT and Spearman correlation analysis, and the correlation between EDNRA and TMB, MSI, IC50, and immune checkpoints was examined.

**Results:** EDNRA expression was significantly higher in STAD than in normal tissues (*P* < 0.001) and associated with worse overall survival (OS). EDNRA expression was significantly associated with T stage, histological type, histologic grade, and TP53 status. Cox regression analysis revealed that primary therapy outcome, age, tumor status, and EDNRA were independent prognostic factors for OS. Multivariate analysis revealed that EDNRA expression, tumor status, age, and primary therapy outcome influenced patient prognosis. GSEA was significantly enriched in several pathways and biological processes, which include Immunoregulatory, Hedgehog, WNT, PI3K-AKT.NK cells, Tem, macrophages, and mast cells were substantially positively correlated with EDNRA expression in the STAD microenvironment. Notably, high EDNRA expression may promote M2 macrophages to block PD-1-mediated immunotherapy and induce immunosuppression. In addition, patients with high expression of EDNRA might be resistant to the treatment of several anti-tumor drugs.

**Conclusion:** Our results suggest that EDNRA was closely related to clinicopathologic characteristics, poor prognosis, and promoted macrophage differentiation and synergistic role in immunosuppression.

## Introduction

Stomach adenocarcinoma (STAD), originates from the gastric mucosal epithelium and is the most common type of gastrointestinal cancer and accounts for 95% of gastric malignancies. Most patients with gastric cancer are in the advanced stages when diagnosed, missing operation chance 1. Clinical immunotherapy, represented by PD‐1/PD‐L1, has led to significant improvements in patient outcomes for STAD 2. However, the molecular and clinical heterogeneity of STAD means that PD-1 blockade response varies from person to person. Therefore, it is imperative to ascertain the molecular mechanism and identify more powerful prognostic biomarkers for improving the efficacy of STAD immunotherapy 3.

The endothelin receptors (EDNRA and EDNRB) are guanine-nucleotide-binding (G) proteins and are activated by endothelins, which are 21-amino peptide agonists 4. Both endothelin receptors are widely expressed in the human body. The Endothelin Receptor Type A (EDNRA) encodes the receptor for endothelin-1, a peptide located primarily in the vascular smooth muscle where it functions in vasoconstriction and cell proliferation. Previous studies have shown that EDNRA is an epithelial-mesenchymal transition (EMT) related gene and regulates a chemoresistant phenotype 5, 6. Several studies have reported that EDNRA was closely related to the occurrence and development of some tumors, including bladder cancer, renal cell carcinoma (RCC), osteosarcoma, and ovarian cancer 7-10. Wei 11 showed that EDNRA is overexpressed in gastric cancer cells compared with normal gastric cells (P < 0.01). EDNRA is the downstream target gene of microRNA-200C, microRNA-200C knockdown, showed that EDNRA can significantly inhibit the proliferation and invasion ability and promote apoptosis in gastric cancer cells. The latest research suggests that high EDNRA expression was correlated with advanced gastric cancer 12. However, the specific mechanism through which EDNRA regulates the occurrence and development of gastric cancer, and its clinical and immune value in gastric cancer, require further clarification.

To understand the role of EDNRA in STAD pathogenesis, we assessed EDNRA expression levels in STAD between different cancers and adjacent normal tissues using data from The Cancer Genome Atlas (TCGA) database. We analyzed the correlation between EDNRA expression level and the clinicopathological factors. The discrimination value of EDNRA was assessed using receiver operating characteristic (ROC) curve analysis. Survival analysis and prognostic significance were validated using Kaplan-Meier and Cox regression methods. The EDNRA-related biological pathways and processes involved in STAD were identified by differential and gene expression analysis and Gene-Set Enrichment Analysis (GSEA). EDNRA protein levels and their clinical value were assessed and confirmed using immunohistochemistry cohort analysis. The correlation between EDNRA expression and immune characteristics was determined to explore the possible mechanism of EDNRA in STAD oncogenesis and the inhibitory effect of immunotherapy.

## Materials and Methods

### Data acquisition and Pan-cancer analysis of EDNRA

We collected patients with STAD from the Cancer Genome Atlas (TCGA) database (https://cancergenome.nih.gov/, accessed July 1, 2018) 13. Normalized transcripts per million reads (TPM) expression data for EDNRA from TCGA Pan-cancer (The Cancer Genome Atlas) 14 and GTEx datasets (2013) 15 were downloaded from the UCSC XENA dataset (http://xena.ucsc.edu/), which included 31 types of tumors and relevant normal tissues. Owing to the application of open access data extracted from TCGA, this research does not require additional approval by an ethics committee. We performed the Wilcoxon rank-sum test to compare EDNRA expression in different cancers and paired normal tissue samples.

### RNA-sequencing data, differential expression, and ROC curve analysis

RNA-seq data were downloaded from UCSC XENA in GTEx TPM format of GTEx were downloaded from UCSC XENA and processed by the Toil project 16. The Wilcoxon rank-sum test was used to compare EDNRA expression in normal TCGA and GTEx samples and tumor samples of TCGA. We collected 32 adjacent normal samples and 375 STAD tumor samples from the TCGA database. The Wilcoxon rank-sum test was used to compare EDRNA expression in normal and tumor samples. We utilized the RNA-seq data of 375 tumors for further association analysis. Unavailable or unknown clinical features in 375 patients were regarded as missing values. The data are summarized in **Table 1.** ROC curve analysis was used to assess the effectiveness of single gene expression values in distinguishing tumor and normal samples, and the area under the curve (AUC) was used as a quantitative evaluation index 17. P < 0.05 was considered statistically significant.

### Correlations between EDNRA expression and clinicopathologic characteristics in STAD patients

We analyzed the correlation between EDNRA expression level and the clinicopathological characteristics of STAD in TCGA by χ^2^-test, Fisher's exact test, Kruskal-Wallis rank-sum test, Wilcoxon rank-sum test, and logistic regression. The χ^2^-test, Fisher's exact test, and logistic regression were performed based on EDNRA high and low classification. The clinicopathological characteristics included TNM stage, pathologic stage, histological type, histological grade, primary therapy outcome, residual tumor, tumor status, TP53 status, and PIK3CA status. 'Exact' means that the Fisher's exact test was used.

### Univariate and multivariate Cox regression analysis of TCGA STAD cohorts and survival analysis

We used univariate and multivariate Cox regression analysis to identify the predictive value. It was performed to compare the effects of EDNRA expression on survival with other clinical traits (TNM stage, pathologic stage, histological type, histological grade, primary therapy outcome, residual tumor, race, gender, Anatomic neoplasm subdivision, reflux history, antiviral treatment, barrettes esophagus, tumor status, TP53 status, PIK3CA status, and EDNRA expression). The TNM staging was conducted according to the International Union Against Cancer (7^th^ edition) 18, in which T represents the range of primary tumors, N represents the presence and extent of regional lymph node metastasis and its scope, M represents the presence or absence of a distant transfer. Accordingly, variables with P < 0.05 in univariate Cox models were then put into multivariate Cox regression analysis. HR with a 95% confidence interval (CI) was used to estimate the hazard risk of individual factors. Kaplan-Meier analysis was used to calculate the difference in overall survival (OS) between high and low EDNRA expression groups in the survival package with a two-sided log-rank test. Five of these patients with missing survival data were excluded from the following analysis. The TCGA STAD cohort was stratified into the high and low EDNRA expression groups. The median expression of EDNRA was selected as the cut-off value.

### Univariate and multivariate Cox regression analysis of the validation cohort

Study protocols were approved by the Ethics Committee of China Medical University (AF-SOP-07-1.1-01), and all participants provided written informed consent which included they have the right to withdraw from the experiment in the course of the experiment. Primary tumor samples were collected from 100 patients with gastric cancer undergoing surgery at The First Affiliated Hospital of China Medical University between 2010-2012. Patients who also underwent neoadjuvant radio- and/or chemotherapy were excluded. The TNM staging was conducted according to the International Union against Cancer (7^th^ edition) 18. The OS was measured from the date of diagnosis to the date of death or last follow-up. Univariate and multivariate Cox regression analyses were performed using SPSS statistical software (version 23.0.0). Clinical traits with P < 0.05 in univariate Cox models were then selected to perform the multivariate Cox regression analysis.

### The clinical validation cohort collection and inclusion criteria

We collected a clinical validation cohort from the First Affiliated Hospital of China Medical University between 2010-2012, which consisted of 100 STAD patients tumor specimens and 20 adjacent non-tumor tissue specimens Study protocols were approved by the Ethics Committee of The First Affiliated Hospital of China Medical University. All participants provided written informed consent. Patients diagnosed with gastric cancer without other serious diseases were enrolled in the study. During surgery, 100 samples of tumor tissue, peritumoral tissue (within 3 cm of the tumor edge), and gastric normal tissue (3 cm from the tumor edge) were collected from the 100 patients and stored at -80 °C for future use. The inclusion criteria were used as follows: (1) patients pathologically confirmed with gastric cancer; (2) patients subjected to surgery; (3) patients aged 18-80 years. The exclusion criteria included receiving neoadjuvant chemotherapy or radiotherapy, remnant gastric cancer, and postoperative death within 3 months. The pathological diagnoses and classifications were estimated according to the AJCC Cancer Staging Manual (7^th^ edition) 18. Survival follow-up data were noted by telephone or medical records.

### Immunohistochemistry and survival analysis

Immunohistochemistry of EDNRA was performed on the validation cohort. All tissue specimens were fixed in neutral formaldehyde, embedded in paraffin, and sectioned (thickness, 4 μm). The streptavidin-peroxidase immunohistochemical method was used to enhance staining intensity. Tissue sections were incubated at 4 °C overnight with anti-EDNRA (1:100) (DF4923; rabbit anti-human; polyclonal; Affinity Biosciences, USA). Finally, samples were lightly counterstained with hematoxylin, dehydrated in alcohol, and mounted. Two pathologists, blinded to the clinical data, independently scored the slides in each sample by evaluating the staining intensity and percentage of stained cells in representative areas. The immunohistochemical staining intensity details were described in our previous study 19. The slides were analyzed by standard light microscopy. The staining-based expression levels were be divided into four categories: positive, moderate, low, and negative by the scoring system. The staining intensity was scored as 0 (negative), 1 (weak), 2 (moderate), or 3 (strong). The percentage of cells stained was scored as 1 (1-25%), 2 (26-50%), 3 (51-75%), or 4 (76-100%). A final combined score of 0-12 was obtained by multiplying the intensity and percentage scores. Patients were classified into high or low EDNRA protein expression groups based on median expression scores. Specimens with scores of > 3 were considered EDNRA-positive and those with scores > 5 indicated strong positive expression. The t-test (Two-tailed) was used to compare EDNRA protein expression between tumor and non-tumor tissue specimens, and clinicopathologic characteristics between high and low EDNRA expression groups were compared using the Pearson χ2 test. A semiquantitative grading system (H-score) was used to compare the immunohistochemical staining intensities of EDNRA, The histochemistry score (H-SCORE) was calculated to assess the expression level of EDNRA based on the staining intensity and the positive cell ratio as follows: H-SCORE = (percentage of cells with weak staining × 1) + (percentage of cells with medium staining × 2) + (percentage of cells with strong staining × 3). To assess the prognostic value of EDNRA protein expression prognostic value, OS differences in high and low EDNRA staining H-SCORE expression groups were determined using Kaplan-Meier survival analysis (as provided in the survival package) in conjunction with the Wilcoxon log-rank test. P-values less than 0.05 were considered significant in all tests.

### Differential expression analysis and GSEA analysis

TCGA STAD samples were divided into high-and-low expression groups based on median EDNRA expression levels, and differential expression analysis was conducted between these groups using the Limma package, and heat maps and volcano maps were generated high expression and low expression groups of EDNRA. The results were presented in the form of the heat map and the volcano map. These analyses were processed with the Limma package (version: 3.40.2). A P value of 0.05 and log2(1.5) was set as the thresholds for significantly differential expression. To further reveal the underlying function of EDNRA in STAD, differently expressed genes (DEGs) were analyzed by Gene set enrichment analysis. Gene set enrichment (http://software.broadinstitute.org/gsea/index.jsp) (GSEA) analysis used gene sets and characteristics that had been a prior associated with various diseases or pathways to provide the biological application to the interesting samples 20. We performed GSEA to elucidate the significant function and pathway differences between the low and high expression EDNTA groups using the R package ClusterPorfiler (http://bioconductor.org/packages/release/bioc/html/clusterProfiler.html) in **Figure 5** 21 (version 3.8.0). We used the Molecular Signature databases (MSigDB) collections (v6.13) as the reference gene set.False discovery rate (FDR) < 0.25, adj. P-value < 0.05 and |NES| > 1 were considered significantly enriched.

### Immune infiltration analysis by ssGSEA

We applied the single-sample Gene Set Enrichment Analysis (ssGSEA) method using the GSVA package (http://www.bioconductor.org/packages/release/bioc/html/GSVA.html). Using R (v.3.6.2), the relative tumor infiltration levels of 24 immune cell types was quantified by interrogating the expression levels of genes in the published signature gene tables 22. The signatures included a set of different adaptive and innate immune cell types. Spearman correlation was adopted to explore the association between EDNRA expression and immune cell infiltration level quantified by ssGSEA in STAD. P< 0.05 and |R| ≥0.4 were considered to be correlated. The Wilcoxon rank-sum test was used to analyze differences in immune cell infiltration levels between the groups with high and low EDNRA expression.

### Immune infiltration analysis in the TIMER database

TIMER (https://cistrome.shinyapps.io/timer/) 23 is a comprehensive online website for the systematic investigation of immune infiltration over various malignancy types. The infiltration levels of six different immune cells, CD8+ T cells, B cells, CD4+ T cells, Macrophages, Neutrophils, and Dendritic cells are statistically assessed and used for pathological estimations. We explored the correlation between EDNRA expression and immune cell infiltration in STAD. The survival module was used to draw Kaplan-Meier plots for immune infiltrates and the relationship between EDNRA expression and the survival plots was assessed. Using the correlation module, we systematically investigated the correlation between EDNRA expression and representative biomarkers of M1 and M2 macrophages, and tumor-associated macrophages (TAMs). Finally, we compared immune cell infiltration levels in samples with somatic EDNRA copy number alterations in STAD (P-Value < 0.05).

### CIBERSORT

To estimate the relative abundance of tumor-infiltrating immune cells in STAD and the correlation with EDNRA expression, we used CIBERSORT (https://cibersort.stanford.edu/) 24 to calculate the proportions of immune cell types in low and high EDNRA expression groups. This process identified 22 sorted immune cell subtypes (LM22) as the gene signature and was evaluated using the CIBERSOFT^1^ R package.

### TISIDB

TISIDB (http://cis.hku.hk/TISIDB/index.php) 25 is a database for analyzing tumor-immune system interactions. TISIDB was used to analyze EDNRA gene expression in STAD immune subtypes, including wound healing (C1), IFN-γ dominant (C2), inflammatory (C3), lymphocyte deplete (C4), immunologically quiet (C5), and TGF-β dominant (C6) subtypes. Additionally, we investigated the EDNRA expression levels in different STAD molecular subtypes.

### The correlation between EDNRA and immune checkpoint, microsatellite instability (MSI), tumor mutational burden (TMB), and IC50

TMB 26 is defined as the total number of somatic mutations identified per coding area of the target sequence. TMB has been widely used to explore the number of mutations in tumors and is a quantifiable biomarker for predicting immunotherapeutic response. Therefore, we separately evaluated the TMB of different tumor types and analyzed the correlation between EDNRA expression and TMB using the Spearman rank correlation coefficient. MSI 27 is a type of hypermutation caused by defects in the mismatched DNA repair system. The correlation between EDNRA expression and MSI was analyzed using the Spearman correlation coefficient. Pan-cancer drug resistance expression profiles were downloaded from the GDSC website 28, and drug IC50 information and EDNRA expression profiles were analyzed by Spearman correlation analysis. The correlation between the top 6 chemotherapy drugs and EDNRA are shown as scatterplots by Spearman rank correlation analysis. These results show that these chemical cancer drugs have the potential to be the EDNRA-molecular target drug **(Figure 10)**.

### Statistical analysis

R (v.3.6.2) was used for all statistical analyses. The Wilcoxon rank-sum test was used to compare EDNRA expression in the TCGA and GTEx normal samples with that in the TCGA tumor samples. The χ^2^-test, Fisher's exact test, Kruskal-Wallis rank-sum test, Wilcoxon rank-sum test, and logistic regression were performed to evaluate correlations between EDNRA expression and clinicopathologic characteristics of STAD patients. Kaplan-Meier and univariate and multivariate Cox regression analyses were used to validate the survival and prognostic significance and ROC curves were obtained using the “pROC” package 17. R package clusterPorfiler was used to elucidate the expressive function and pathway difference between the low and high EDNRA expression groups. FDR < 0.05, adj. P-value < 0.05 and|NES| > 1 were considered significantly enriched. The relationship between EDNRA and immunohistochemistry parameters was assessed and visualized using GraphPad Prism (version 7.0). We used one-way ANOVA to conduct multiple-group comparisons, and we used the t-test to conduct two-group comparisons. Spearman correlation was used to explore the relationship between EDNRA expression and immune cell infiltration levels and IC50. P < 0.05 and |R| ≥0.4 were considered to be correlated. The Wilcoxon rank-sum test was used to analyze the differences in immune cell infiltration levels between the groups with high and low EDNRA expression. P < 0.05 was considered statistically significant in all tests.

## Results

### EDNRA is a promising biomarker for STAD

**Figure 1:**
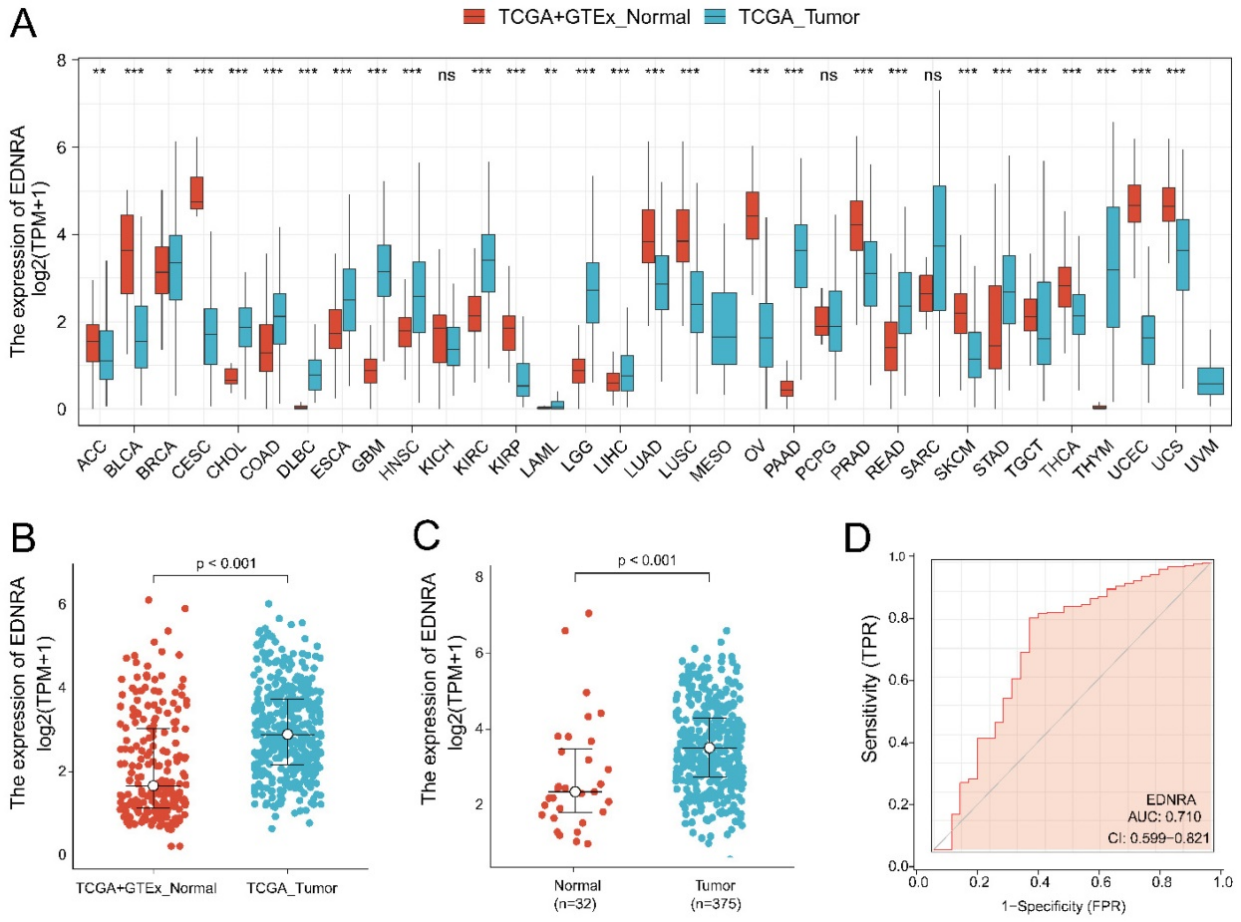
** EDNRA expression levels in different types of human cancers and STAD, the diagnostic value. (A)** The expression of EDNRA in different types of human cancers. **(B)** The expression of EDNRA between normal (TCGA + GTEx) and tumor tissues (TCGA). **(C)** The expression of EDNRA between 375 STAD tissues and 32 adjacent normal tissues from TCGA. **(D)** The ROC curves of EDNRA in STAD.

Analysis of all TCGA tumors revealed that EDNRA was significantly overexpressed in STAD samples (P < 0.001) (**Figure 1A-B**). Then, we examined EDNRA expression in 375 cancer tissues and 32 adjacent normal tissues in TCGA. EDNRA was more prominently overexpressed in cancer tissues than in adjacent normal tissues (P < 0.001) (**Figure 1C**). We performed a ROC curve analysis of data from patients with STAD and healthy people to assess the discrimination value of EDNRA. The AUC was 0.710, indicating that EDNRA may be an effective diagnostic molecule for STAD (**Figure 1D**).

### Correlations between EDNRA expression and clinicopathologic characteristics in patients with STAD

**Figure 2:**
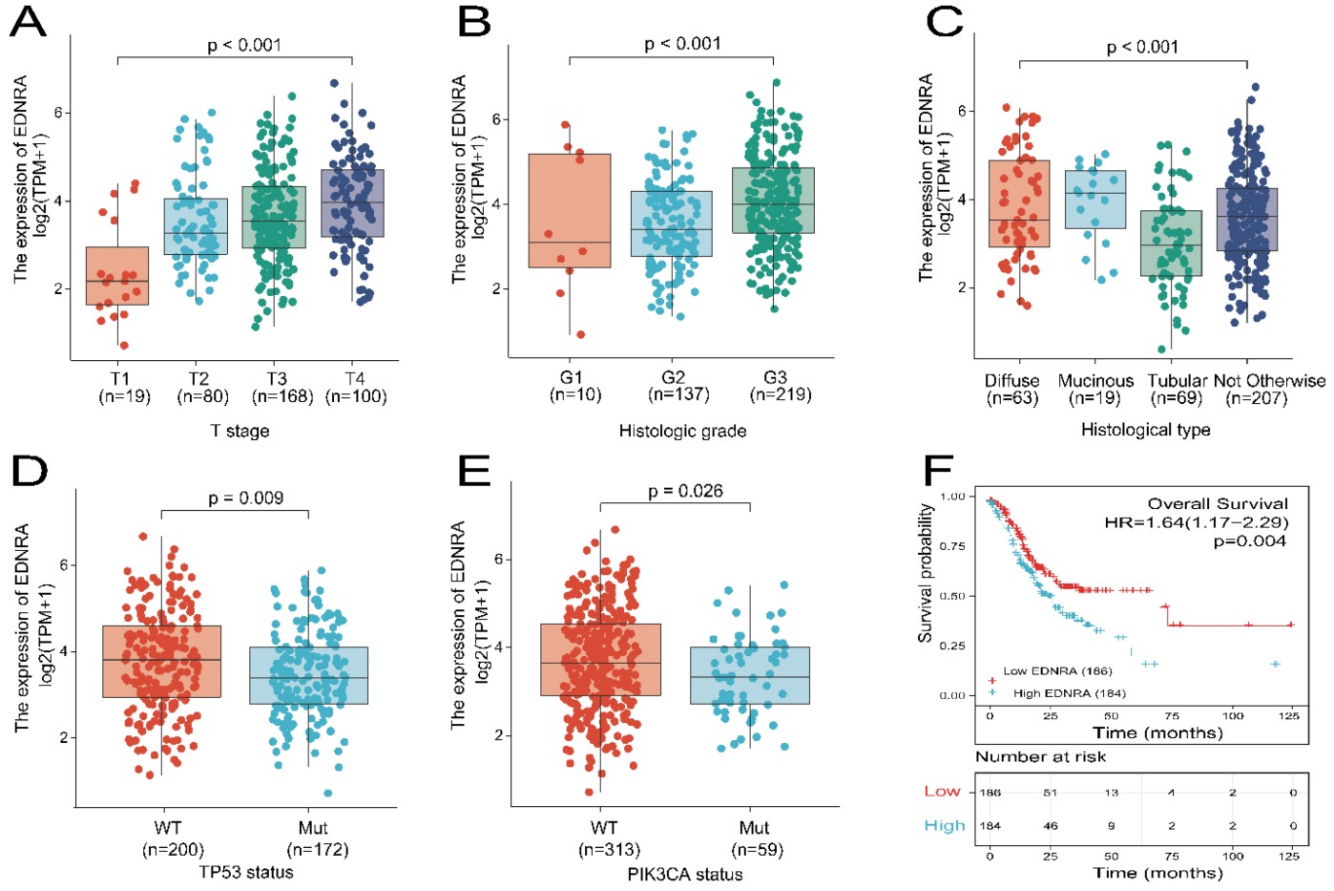
** Expression of EDNRA correlated with clinicopathologic characteristics of STAD patients and overall survival analysis. (A)** Expression of EDNRA correlated significantly with T stage **(B)**, histological type **(C)**, histological grade **(D)**, TP53 status **(E)** PIK3CA status. **(F)** The correlation between EDNRA expression and overall survival (OS), as shown in the Kaplan-Meier survival plot.

The data were collected from TCGA and included 375 primary tumors with clinical and gene expression data (**Table 1**). The patients were divided into two groups with low (188 cases) and high (187 cases) EDNRA expression in STAD. There were 134 females (35.7%) and 241 males (64.3%) in the cohort. The percentage of patients younger than 65 years was 43.7% TNM stage data showed that 246 (68.9%) of 357 cases had regional lymph node invasion, and 25 (7%) of 355 cases had distant metastases. Histologically, 63 (16.9%) cases were diffuse type, 19 (5%) cases were mucinous type, 207 (55.3%) cases were not otherwise specified, 63 (18.4%) cases were Tubular type. For histological grade, 10 (2.7%) cases were G1, 137 (37.4%) cases were G2, and 219 (59.8%) cases were G3. Assessment of TP53 status revealed that 172 (46.2%) cases were had mutant TP53, and 200 (53.8%) were wildtype. PIK3CA status assessment revealed 59 (15.9%) cases were mutant, and 313 (84.1%) cases were wild-type. EDNRA expression was significantly associated with T stage (P < 0.001), histological type (P = 0.017), histologic grade (P = 0.018), TP53 status (P = 0.016), using χ^2^-test or Fisher's exact test. EDNRA expression did not significantly differ were in respect to age, gender, N stage, M stage, and PIK3CA status (P > 0.05). The relationship between EDNRA expression and clinicopathologic characteristics is shown in **Figure 2A-E**. EDNRA expression in STAD was significantly associated with T stage (P < 0.001), histological type (P < 0.001), histologic grade (P < 0.001), TP53 status (P = 0.009), and PIK3CA status (P = 0.026) of STAD. Logistic regression analysis was utilized to assess the relationship between clinicopathological characteristics of patients with STAD and the classification of EDNRA expression as high or low (**Table 2**). These results suggest that EDNRA expression is significantly related to T stage (P < 0.001), histological type (P = 0.028), histological grade (P = 0.005), and TP53 status (P = 0.012). Notably, high expression of EDNRA was accompanied with less TP53 mutant status.

### Association between OS and clinicopathological characteristics in TCGA and STAD cohorts using univariate and multivariate Cox regression

Kaplan-Meier analysis using the 'survminer' package was used to assess the prognostic value of EDNRA in STAD. OS was poorer in patients with high EDNRA expression than in those with low EDNRA expression (HR = 1.64 (1.17-2.29), P = 0.004, **Figure 2F**). Univariate and multivariate analyses using the Cox regression model from the TCGA datasets were performed to identify whether EDNRA expression is an independent prognostic factor for OS (**Table 3**). Variables with P < 0.05 in the univariate Cox regression analysis were included in the multivariate Cox regression. The variables that met this threshold were T stage (HR=1.719, P = 0.011), N stage (HR=1.925, P = 0.002), M stage (HR=2.254, P = 0.004), pathologic stage (HR=1.947, P < 0.001), primary therapy outcome (HR=0.237, P < 0.001), residual tumor (HR=3.445, P < 0.001), age (HR=1.620, P = 0.005), tumor status (HR=5.420, P < 0.001), and EDNRA (HR=1.638, P = 0.004). Multivariate Cox regression analysis identified, primary therapy outcome (HR=0.522, P = 0.018), age (HR=1.734, P = 0.017), tumor status (HR=3.642, P < 0.001), and EDNRA (HR=1.566, P = 0.049) as independent prognostic factors in OS (P < 0.05). In STAD validation cohorts, only EDNRA served as an independent predictive factor of OS after multivariate Cox regression analysis (**Table 4**). In summary, this evidence indicates that EDNRA expression serves as an independent biomarker of STAD prognosis.

### Immunohistochemistry analysis of EDNRA

**Figure 3:**
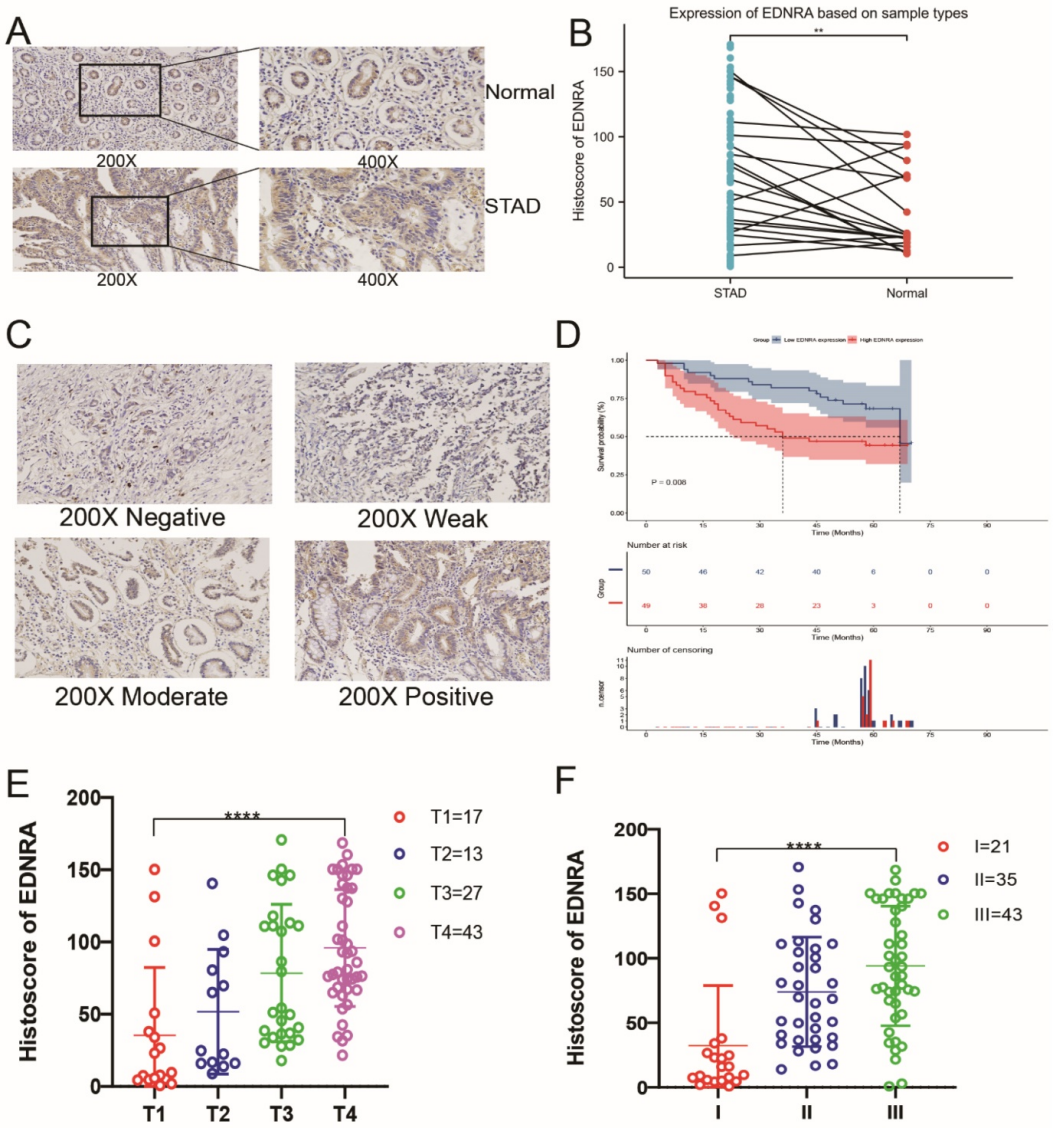
** The preliminary immunohistochemistry analysis and clinical analysis of EDNRA in STAD. (A)** Representative Immunohistochemistry image of EDNRA and subcellular staining localization in STAD and adjacent non-tumor tissue specimens. **(B)** The expression of EDNRA in STAD tissue was higher than adjacent non-tumor tissue (P<0.01). **(C)** The representative staining of EDNRA in different staining classes. **(D)** Overall survival analysis revealed that high EDNRA expression indicates a poor prognosis (P = 0.008). **(E)** The analysis of staining expression level correlated significantly with the T stage. **(F)** The analysis of staining expression level correlated significantly with Histological grade. I, Grade 1; II, Grade 2; III, Grade 3 (P<0.001).

To determine the differences between EDNRA protein expression levels in STAD and adjacent non-tumor tissue, and whether EDNRA protein expression correlates with other clinicopathologic characteristics, we utilized this validation cohort to investigate EDNRA expression in adjacent normal tissues and STAD by immunohistochemistry. The expression of EDNRA in STAD tissue was higher than adjacent non-tumor tissue (P<0.01), and EDNRA was mostly expressed in the cytoplasm but also stained positive in normal tissues and cancer cells (**Figure 3A-B**). To analyze the effect of the expression level of EDNRA on the prognosis of STAD, we performed Kaplan-Meier survival analysis based on histoscore. The results showed that patients with relatively high EDNRA expression had a poorer survival rate than those with low EDNRA expression (**Figure 3C-D**). To compare the relative EDNRA expression in different pathological grades and T classification, we analyzed the validation cohorts using one-way ANOVA with a post-hoc Tukey's test. The results showed that the higher EDNRA protein expression was associated with higher STAD pathological grade and T classification (P<0.001). These results confirmed that EDNRA serves as a driving force for promoting the development of STAD and worsening the patient's prognosis (**Figure 3E-F**).

### The differentially expressed genes in correlation with EDNRA and GSEA analysis in STAD

**Figure 4:**
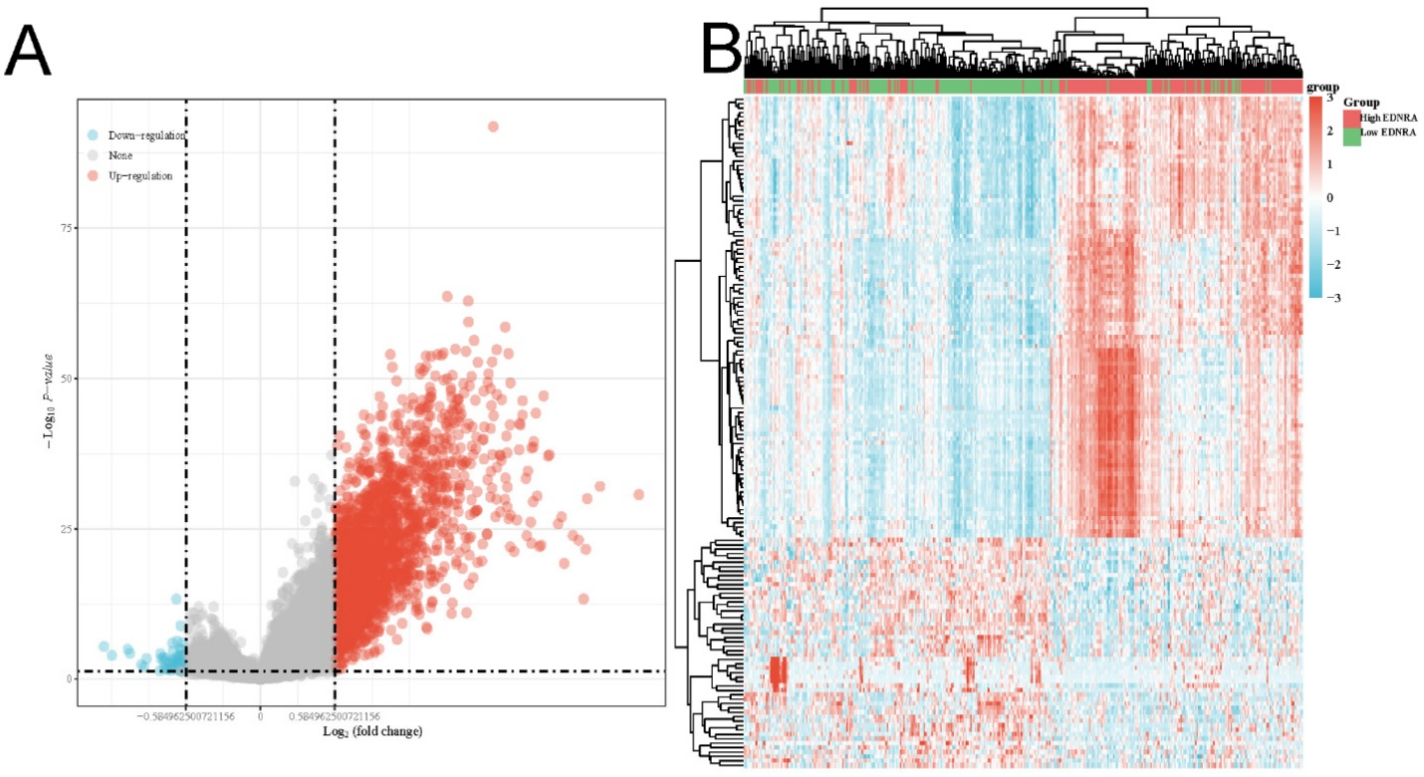
** The differentially expressed genes in correlation with EDNRA in STAD.** The volcano plot **(A)** and the heatmap **(B)** shown the differential expression genes between the high and the low expression of the EDNRA group.

**Figure 5:**
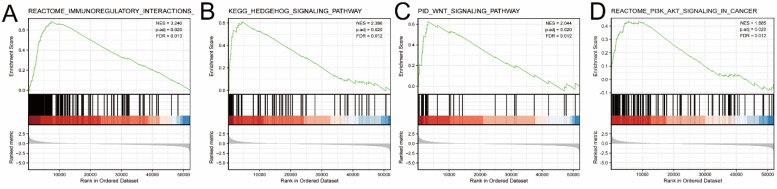
** GSEA analysis of EDNRA in TCGA-STAD data.** Several pathways and biological processes were differentially enriched in ENDRA-related STAD, GSEA showed the top-four pathways associated with EDNRA. **(A)** Immunoregulatory, **(B)** Hedgehog signaling pathway, **(C)** WNT signaling pathway, **(D)** PI3K-AKT signaling pathway ES, enrichment score; NES, normalized ES; adj. *P*, adjust *P-*value; FDR, false discovery rate.

As shown in the volcano plot (**Figure 4A**), 2,124 genes (Red dots) showed significant positive correlations with EDNRA, whereas 5,2 genes (Blue dots) showed significant negative correlations (false discovery rate [FDR] < 0.01). Hierarchical clustering analysis of mRNAs, which were differentially expressed between high and low expression groups of EDNRA, was shown as a heatmap (**Figure 4B**). This result suggests a widespread impact of EDNRA on the transcriptome. Four signaling pathways, including Immunoregulatory, Hedgehog, WNT, and PI3K-AKT signaling pathways showed significantly differential enrichment in EDNRA high expression phenotype. Immunoregulatory (NES = 3.246, adj. P = 0.020, FDR = 0.013, **Figure 5A**). Hedgehog (NES = 2.386, adj. P = 0.020, FDR = 0.012), WNT (NES = 2.044, adj. P = 0.020, FDR = 0.012) and PI3K-AKT (NES = 1.88, adj. P = 0.020, FDR = 0.012, **Figure 5B-D**). Biological process immunoregulatory also showed significantly differential enrichment in EDNRA high expression phenotype. It indicated that EDNRA might be related to these signaling pathways, biological processes, and the potential role of EDNRA in the immune regulation of STAD.

### The correlation between EDNRA expression and abundance of the immune infiltration

**Figure 6:**
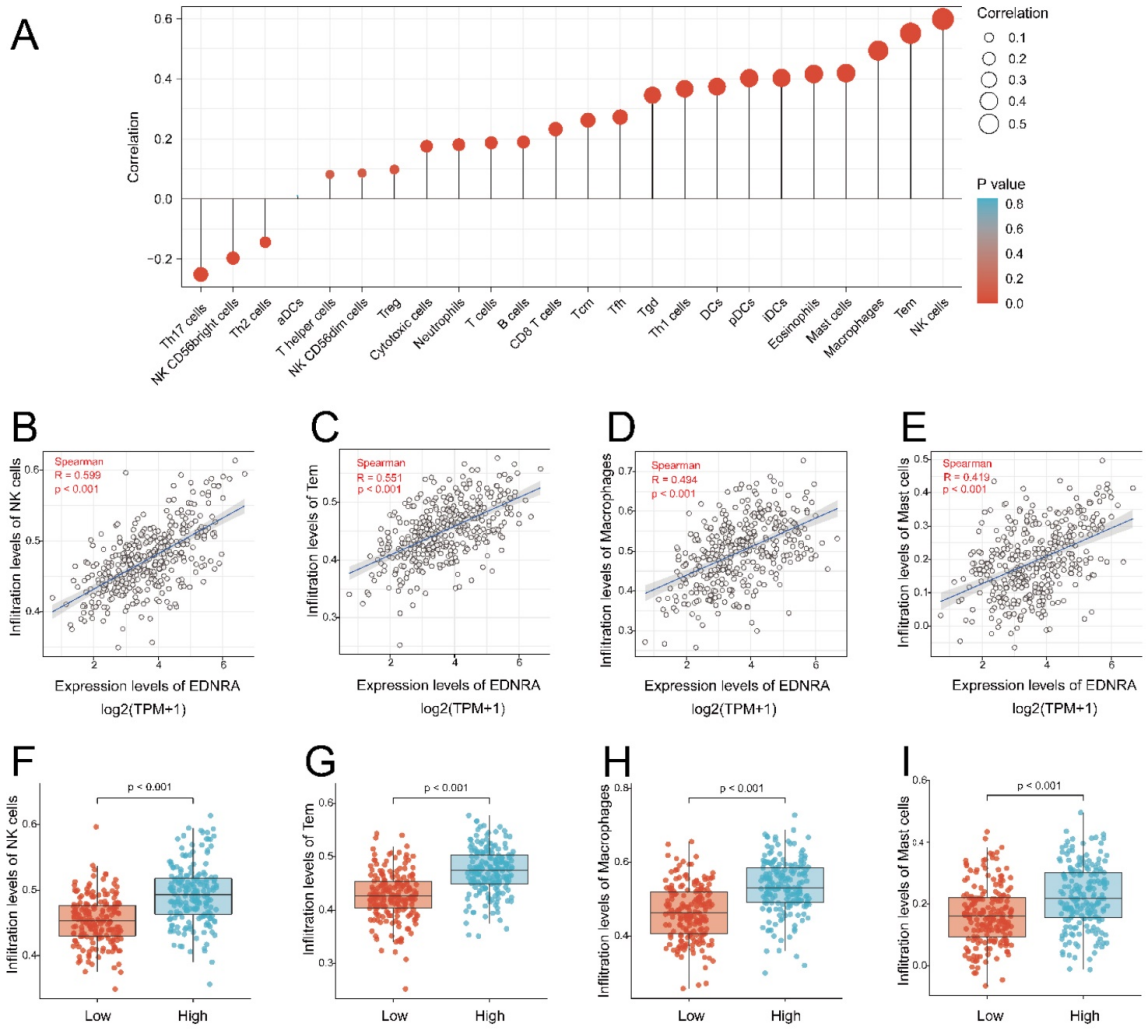
** Correlation of EDNRA expression with immune infiltration level in STAD by ssGSEA. (A)** The association between the EDNRA and 24 subtypes of immune cells level in STAD **(B-E)** Correlations between EDNRA expression and immune infiltration levels of NK cells, Tem, Macrophages, Mast cells. **(F-I)** The immune infiltration level of NK cells, Tem, Macrophages, Mast cells in high and low EDNRA expression groups in STAD.

The result showed the association between the expression level of EDNRA and immune cell infiltration level quantified in STAD. The NK cells, Tem, macrophages, mast cells, eosinophils, iDCs, PDCs, DCs, Thl cells, Tgd, and so on were correlated with EDNRA expression (**Figure 6A**). NK cells (R = 0.599, P< 0.001), Tem (R = 0.551, P< 0.001), macrophages (R = 0.494, P< 0.001), mast cells (R = 0.419, P < 0.001) as shown in (**Figure 6B-E**), were substantially positively correlated with EDNRA expression. The levels of immune cell infiltration (NK cells (P< 0.001), Tem (P = 0.002), macrophages (P< 0.001), mast cells (P< 0.001) were significantly higher in the EDNRA high expression group compared with the low expression group (**Figure 6F-I**).

### The immune infiltration landscape of EDNRA

**Figure 7:**
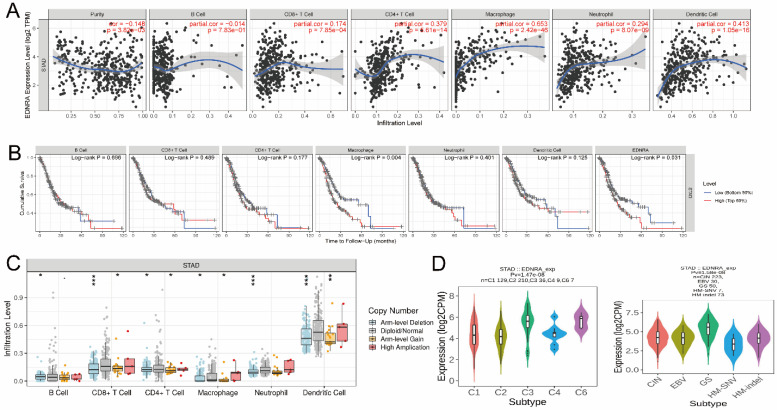
** The immune infiltration landscape of EDNRA. (A)** Correlation between EDNRA expression and abundance of immune infiltration. **(B)** Clinical survival analysis between EDNRA and different immune cell types. **(C)** The relationship between the somatic copy number alterations (SCNA) of EDNRA and abundance of immune infiltration **(D)**. The expression level of EDNRA in different immune and molecular subtypes from TCGA STAD. The P-value Significant: 0 ≤ *** < 0.001 ≤ ** < 0.01 ≤ * < 0.05 ≤ . < 0.1.

It was revealed that EDNRA showed a significant correlation with the abundance of immune infiltration (P < 0.05), (**Figure 7A**). The results confirmed that EDNRA is strongly associated with the macrophage by Timer(cor=0.653), which needs further research. Subsequently, clinical survival mode showed that Macrophages of immune infiltration were statistically significant (P < 0.05) affecting the prognosis of STAD with the synergism of EDNRA (**Figure 7B**) Finally, we use the tool of GISTIC 2.0 by Timer database to clarify the effect of SCNAs, box plots are shown to illustrate the proportions of different immune cell types with the copy number status of EDNRA in STAD (**Figure 7C**). In the analysis of the SCNAs of EDNRA, the deletion of EDNRA led to a lower level of immune infiltration cells. Next, the role of EDNRA expression on STAD immune and molecular subtypes was analyzed by the TISIDB website. Notably, the results demonstrated that the EDNRA expression varied significantly in these molecular subtypes and immune subtypes of STAD (**Figure 7D**). To sum up, the above results indicate that EDNRA may play a complex role in STAD immune microenvironment and show a synergistic role with macrophages to affect STAD prognosis.

### EDNRA is significantly associated with M2 macrophage polarization in STAD

**Figure 8:**
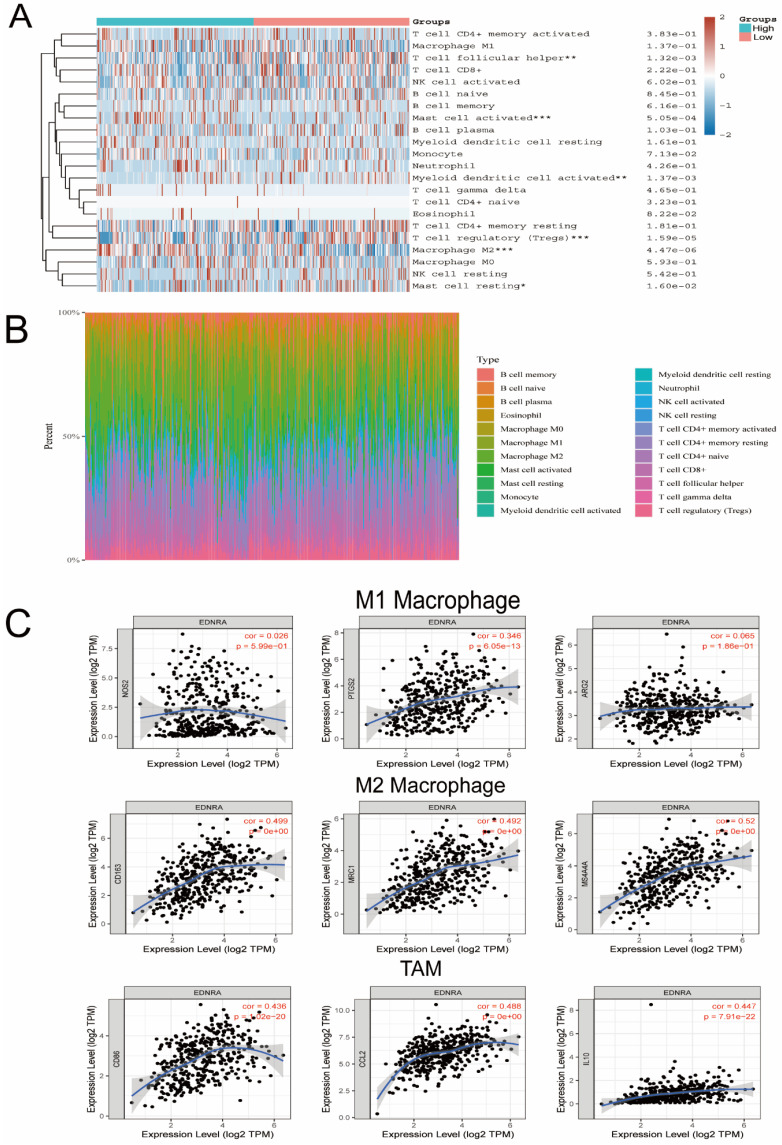
** EDNRA is significantly associated with M2 macrophage polarization in STAD.** The heatmap **(A)** and barplot **(B)** showed the difference of immune cell infiltration based on LM22 between high and low expression groups of EDNRA. **(C)** The correlation analysis of EDNRA expression with macrophage polarization in STAD. Scatterplots of correlations between EDNRA expression and biomarkers of M1 macrophages (iNOS, COX2, and ARG2), M2 macrophages (MRC1, CD163, and MS4A4A), and TAMs (tumor-associated macrophages) (CD86, CCL2, and IL10) are outlined.

CIBERSORT was applied and it revealed that M2 macrophages, Mast cells were significantly upregulated in the high expression group of EDNRA, whereas M1 macrophages showed no significant change (**Figure 8A-B**), which suggested that EDNRA may specifically influence the polarization of M2 macrophages. To further confirm the association between EDNRA and different macrophage subtypes, we systematically investigated the correlation of EDNRA and the markers of M1 and M2 macrophages, as well as TAMs in STAD by TIMER database. The results showed that M1 macrophage markers such as PTGS2 and ARG2 had weak correlations with EDNRA expression, while gene markers of M2 macrophages such as CD163, MRC1, and MS4A4A and TAM markers such as CD86, CCL2, and IL10 had a close correlation with EDNRA (**Figure 8C**). These results suggested that EDNRA may have a potential regulatory role in the polarization of M2 macrophages and the differentiation of macrophages into tumor-associated macrophages (TAMs). Furthermore, Previous studies have reported that Treg cells and M2 macrophages are well known as potent immunosuppressive agents for PD-1 immunotherapy. Given the macrophage cell worsen the prognosis of STAD with EDNRA above, therefore, these results indicate that high EDNRA may promote M2 macrophage polarization and differentiation into TAMs, which contributes to STAD carcinogenesis and immune escape.

### EDNRA is synergistic with the immune checkpoint molecules

**Figure 9:**
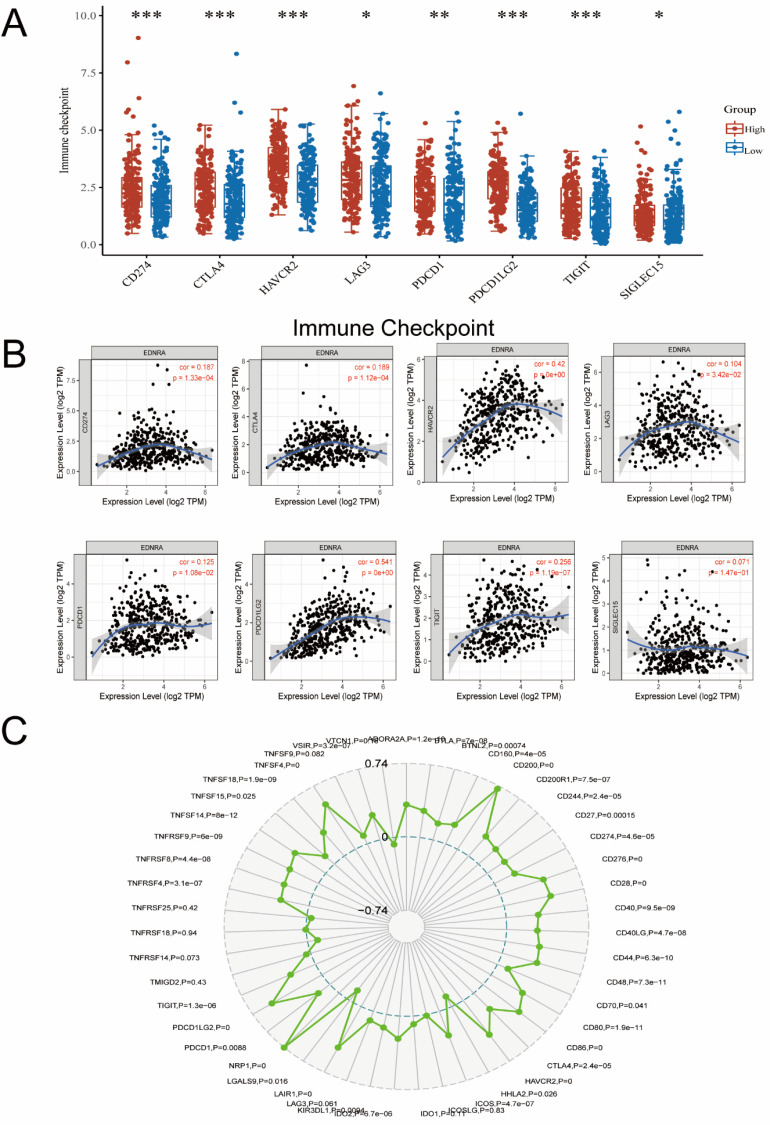
** The correlation between immune checkpoints between the different expressions of EDNRA. (A)** The expression level of classic immune checkpoint between the different expression groups of EDNRA. **(B)** The analysis of correlations among EDNRA with classic immune checkpoint in the stomach (STAD) microenvironment by Timer database. The correlations among EDNRA and CD274, CTLA4, HAVCR2, LAG3, PDCD1, PDCD1LG2, TIGIT, and SLGLEC15 in the STAD microenvironment. **(C)** The radar diagram showed the correlation between EDNRA and novel immune checkpoints in the STAD microenvironment.

Furthermore, we investigated the correlation of EDNRA with Immunotherapy biomarkers for predicting the immunotherapeutic response. Notably, biomarkers that can predict responsiveness to immune-checkpoint blockade are being extensively investigated to further improve precision immunotherapy 29. Recently, Immunotherapy with checkpoint-blocking antibodies targeting CTLA-4 and PD-1/PD-L1 has improved the outlook for patients with a variety of malignancies 30. Considering that the immune checkpoint members play a central role in tumor immune processes, we assessed the correlation between EDNRA and several crucial immune checkpoints in STAD samples. In our study, EDNRA was found to be not only a biomarker of prognosis in STAD but also a bridge correlating M2 macrophage with immune checkpoint members of STAD. In detail, EDNRA was significantly positively correlated with CD200, NRF1 (**Figure 9C**), PDCD1LG2, and HAVCR2 (**Figure 9A, B**) in the STAD microenvironment. On one hand, PDCD1LG2, HAVCR2 were correlated with the STAD microenvironment, and their high expression indicates a suppressive immune status in STAD. On the other hand, NRP1 31 and CD200 32 have been identified as a key novel blockade point combined with PD-1 blockade recently, and their high expression also promotes immunosuppression in the tumor microenvironment 31, 33.

Taken together, the correlations among EDNRA and the related immune markers described above in STAD may indicate a high level of immunosuppression in the STAD microenvironment. Hence, the role and correlation of EDNRA in the STAD microenvironment possibly promote cancer cell growth in STAD and therefore lead to the poor prognosis of STAD patients. EDNRA may be a promising novel therapeutic target to combine with PD-1/PD-L1 blockade.

### The Correlation between EDNRA and Microsatellite instability, TMB and IC50

**Figure 10:**
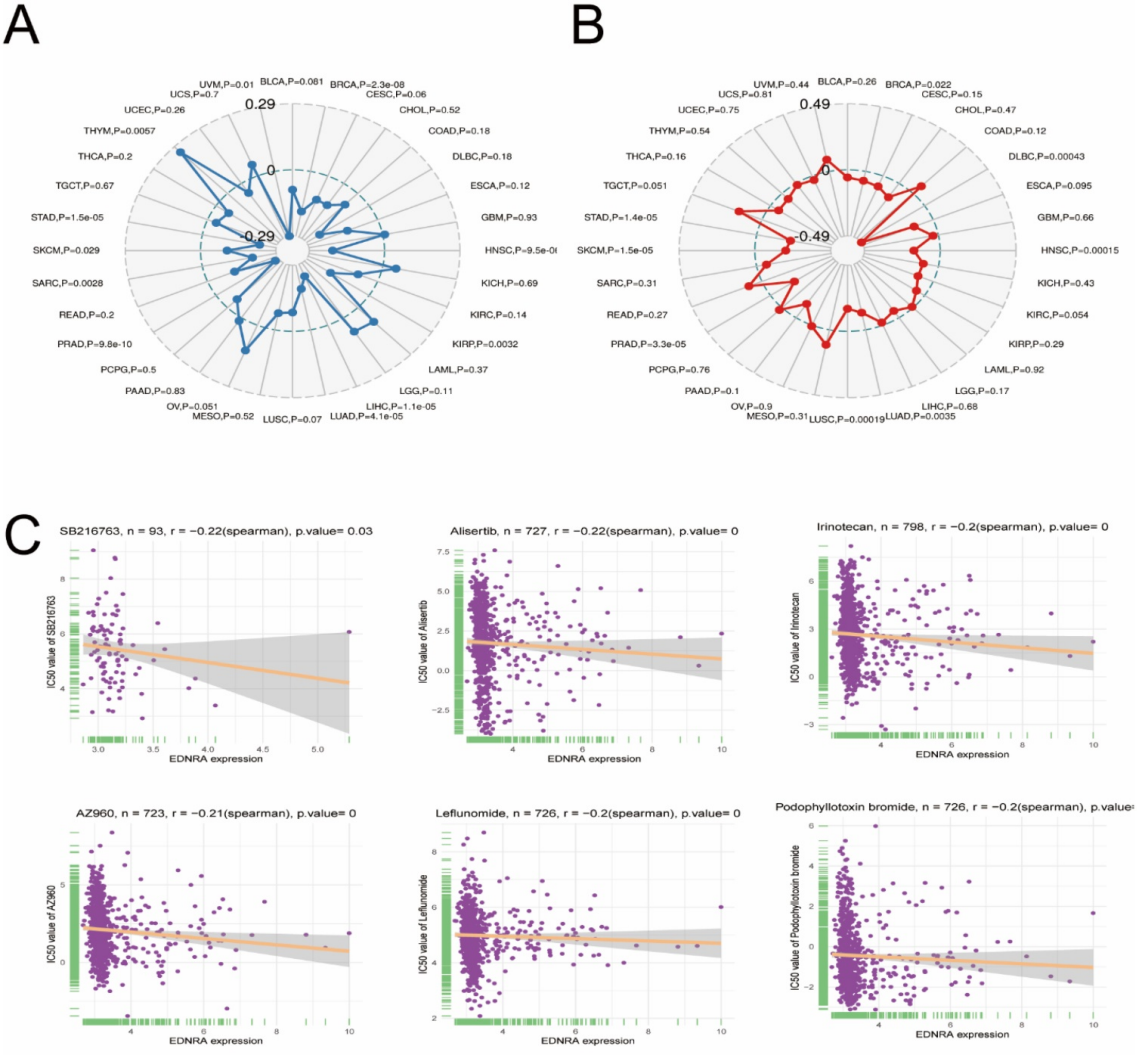
Correlation between EDNRA and Microsatellite instability** (A)**, TMB **(B)**, and IC50 **(C)**.

MSI and TMB are currently the most valuable predictive biomarkers for anti-PD-1 immunotherapy, therefore, we analyzed the correlation between EDNRA and MSI and TMB. Results showed a significant negative correlation between EDNRA expression and MSI in STAD and other tumors. These results significantly revealed a negative correlation between EDNRA and MSI and TMB in STAD (**Figure 10A-B**). These results suggest that the high expression level of EDNRA may indicate the relatively poor PD-1 therapeutic efficacy, which requires further clinical trials to determine.

On the other hand, to identify potential anti-STAD drugs that are associated with the EDNRA, we thus attempted to identify the potentially sensitive and selective chemotherapy drugs using the GDSC drug-sensitivity database. We analyzed drug sensitivity on more than 100 drugs in the GDSC database, then the top six sensitive response drugs were charted by the Spearman correlation analysis (**Figure 10C**).

## Discussion

In this study, we comprehensively explored the clinicopathological significance, expression signature, immunotherapy value, and functional mechanism of EDNRA in STAD. On the one hand, most STAD patients had insidious early symptoms and were already at a very advanced stage when diagnosed, leading to poor prognosis and low survival rates 34. On the other hand, Uncertainties surrounding the efficacy of PD-1 35 compelled us to find a more accurate biomarker combing with PD-1 immunotherapy blockade treatment.

Endothelial receptors show relatively high expression in the cardiovascular system, nervous system, and gastrointestinal tract. They were closely related to cell proliferation, vasoconstriction and relaxation, gastrointestinal motility, and glandular secretion 36, 37. Endothelin-1 (EDN1) was the most potent vasoconstrictor found so far. As the downstream G-protein-coupled receptor of EDN1, the endothelin A receptor (EDNRA) could mediate vascular smooth muscle contraction and proliferation after activation. Previous reports have shown that EDNRA has a significant effect on tumors. Laurberg et al. showed that EDNRA is a potential prognostic marker for bladder patients 7. Pflug 8 found that ET-1 binds with ETA and significantly inhibited paclitaxel-induced RCC cell apoptosis through the PI3K/Akt pathway. The latest research suggests that EDNRA was one of immune-related genes and closely related to tumor infiltration of macrophages in bladder cancer 38. However, the role of EDNRA in gastric cancer was not studied enough.

In the first part of our study, we investigated the expression signature of EDNRA in STAD, including the expression level, discrimination value, prognostic values, and possible mechanism of EDNRA in STAD. In this study, we used mRNA sequencing data obtained from TCGA to explore the analysis of the diagnosis and prognostic value of EDNRA in STAD. Our results showed that EDNRA was overexpressed in STAD samples and may be a potential diagnostic molecule for STAD. Short survival time and poor prognosis were associated with high expression of EDNRA, and its expression was associated with STAD's clinicopathological characteristics (T stage, histological type, histologic grade, TP53 status). Therefore, our analysis suggested that EDNRA could be used as a reference to predict the early diagnosis of tumors and as a potential prognostic marker of STAD. To better explore the clinical role of EDNRA in gastric cancer, we performed the immunohistochemistry experiment validation and relevant clinical cohorts analysis, which revealed that EDNRA expression in gastric cancer tissues is lower than that in adjacent normal tissues and that the high expression of EDNRA indicates with worse prognosis in patients. In contrast, gastric patients with a lower EDNRA expression possess higher overall survival times in our cohorts. In addition, high expression of EDNRA in gastric cancers is associated with the advanced STAD pathological grade and T classification.

To further investigate the function of EDNRA in the occurrence and development of STAD, we performed the differential expression analysis in groups based on the EDNRA expression, the results indicate that the differential expressed genes related to EDNRA were mainly upregulated. then GSEA analysis was carried out and found that Immunoregulatory Hedgehog, WNT, and PI3K/AKT in STAD were significantly enriched especially in EDNRA high expression group. Previous research suggested that EDNRA is associated with the EMT process, ENDRA was an EMT-related gene 6, 39. According to some previous studies, there was a complex network among these pathways in the EMT process. This suggested that EDNRA may mediate the EMT process of gastric cancer through the above pathway, and the specific mechanism should be verified by cell and mechanism experiments. For example, The Hedgehog pathway can directly regulate the expression of EMT-related transcription factors, thus affecting cell EMT 5. Besides, due to the Wnt pathway's activation, a large amount of catenin protein was promoted into the nucleus, which inhibited the E-cadherin/β-catenin complex, weakened the adhesion between cells, and also affected the EMT process of the cells 6. Some research has also confirmed that TGF-β can activate Akt through the PI3K signaling pathway in EMT-producing epithelial cells, thus promoting the occurrence of EMT 8. Our study found that Hedgehog, WNT, and PI3K/AKT signaling pathways in STAD were significantly enriched in the EDNRA high expression group.

In the second part, we mainly focus on the exploration of the immunity value of EDNRA in STAD. Tumor microenvironment immune features have been identified as one of the top ten characteristics 40. Besides, our study revealed that the expression level of EDNRA in STAD was related to immune cell infiltration. The cells with high correlation included NK cells, Tem, Macrophages, Mast cells, suggesting that EDNRA participated in a more complex immune regulatory network. Besides, the Timer analysis also showed that EDNRA was strongly correlated with macrophages. Meanwhile, In the survival curve, the tumor-associated macrophages with high expression of EDNRA significantly affect the STAD patient's prognosis. These results suggested that EDNRA and macrophages played a synergistic role in the occurrence and development of STAD, thus affecting the prognosis of patients. The tumor microenvironment mediated by lymphocytes that infiltrate in the tumor helps tumor cells to escape immune surveillance, and thereby promotes the malignant tumor development and metastasis, while the tumor-associated macrophage (TAM) plays a major role 41. TAM is macrophages infiltrating around the tumor tissue, which has evolved from peripheral monocytes. Under the influence of the tumor microenvironment, various cytokines' secretion plays an important role in the occurrence, metastasis, and invasion of tumors. TAM in tumor tissues transformed into M2-type macrophages in large quantities to promote the occurrence, invasion, and metastasis of tumors and induce EMT 42. For further analysis, we used the CIBERSORT algorithms and revealed that M2 macrophages, Mast cells, and Tregs were significantly upregulated in the high expression group of EDNRA, whereas M1 macrophages showed no significant change, which suggested that EDNRA may specifically influence the polarization of M2 macrophages. Targeting the differentiation of M1-type and M2-type TAM may be an effective means to treat tumors 43. To further confirm the association between EDNRA and different macrophage subtypes, we investigated the correlation between EDNRA and biomarker genes of TAMs and M1 and M2 macrophages. The results indicated that EDNRA has a strong relationship with M2 biomarkers, and TAM markers had moderate relationships while it has a weak relationship with M1 macrophage marker genes. These analyses identified that a high expression level of EDNRA in STAD may promote the polarization of macrophages to M2 macrophages and their eventual differentiation into TAMs, thus prompting an immunosuppressive state on the tumor microenvironment, which eventually promotes the development of STAD.

**Figure 11:**
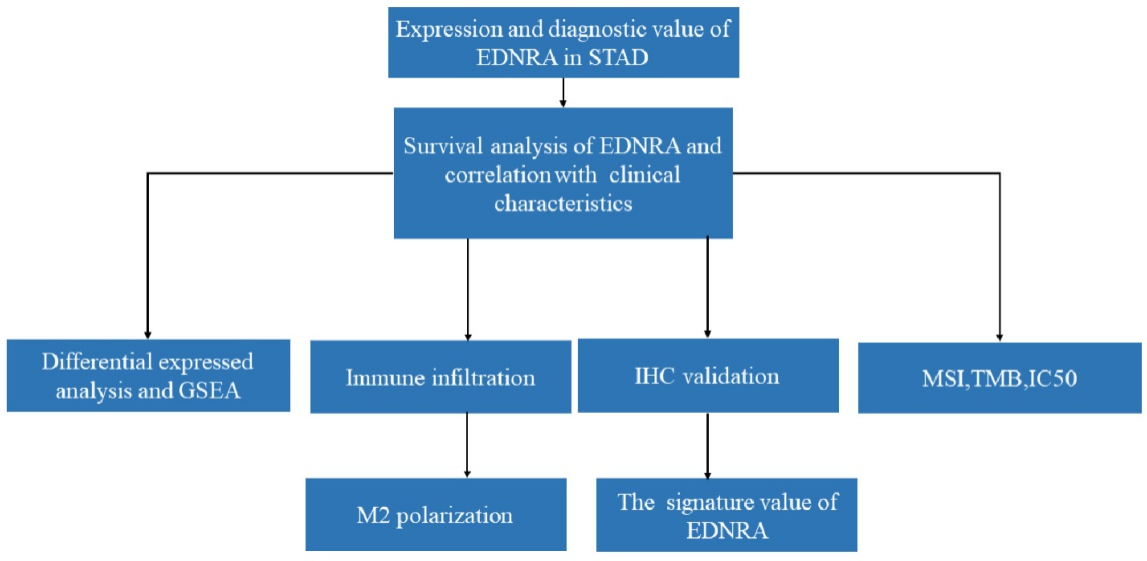
The workflow of a comprehensive analysis of EDNRA is outlined.

Furthermore, previous studies reported that tumor-associated macrophages can deplete anti-PD1 antibodies and therefore negatively interfere with checkpoint inhibition 44. Understanding the balance between tumor-associated macrophages and immune checkpoint blockade may better elaborate the targetable mechanisms behind immune-evasion, specifically for different gastric cancer type 45. In our study, the classic immune checkpoint expression level in the high expression group of EDNRA was generally higher than the corresponding low expression group. To be detailed, EDNRA positively correlated with CD200, NRF1, PDCD1LG2, and HAVCR2 in the STAD microenvironment, therefore, it indicated the EDNRA may play a potential synergistic role with these immune checkpoints to help the tumor realize the immune escape. Future research should focus on exploring multi-checkpoint blockade combined like the PD-1 inhibitor with EDNRA inhibitors for STAD treatment. These findings verified the central role of EDNRA expression in STAD prognosis and tumor immune microenvironment, and shed light on a novel area for further exploration and confirmation (**Figure 11**).

Although our study was the first to explore the comprehensive role of EDNRA in STAD, there are still some limitations of this research, First of all, although the results of our study have been verified by immunohistochemistry clinical samples, the number of cases is small. The second is the cell-level experiment, which should perform to validate the molecular mechanism of EDNRA for STAD and the relevant verification of EDNRA in promoting an immunosuppressive state. In the future, we will continue to conduct further experiments to verify the biological function of the immune mechanism through which it affects the occurrence and development of STAD.

## Conclusions

In summary, we applied integrated bioinformatic approaches, *in vitro*, and clinical analyses to investigate the expression signature, clinical parameters, prognosis value, relevant pathways, relationship with immune infiltration, and correlation with the immune checkpoints of EDNRA in STAD, suggesting that high EDNRA was closely related to clinicopathologic characteristics**,** poor prognosis, and promoted macrophage differentiation and synergistic role in immunosuppression. This new insight might provide a novel direction to explore the pathogenesis of STAD.

## Supplementary Material

Supplementary data.

## Figures and Tables

**Table 1:** Clinical characteristics, the association between EDNRA expression and clinicopathological characteristics in stomach adenocarcinoma (STAD) on TCGA

Characteristics	Low expression of EDNRA n (%)	High expression of EDNRA n (%)	p	test
n	188	187		
**Gender**				
Female	71 (37.8%)	63 (33.7%)	0.474	exact
Male	117 (62.2%)	124 (66.3%)		
**Age**				
≤65	86 (46.2%)	78 (42.2%)	0.493	
>65	100 (53.8%)	107 (57.8%)		
**T stage**				
T1	15 (8.0%)	4 (2.2%)	<0.001	
T2	50 (26.6%)	30 (16.8%)		
T3	88 (46.8%)	80 (44.7%)		
T4	35 (18.6%)	65 (36.3%)		
**N stage**				
N0	54 (29.7%)	57 (32.6%)	0.182	
N1	56 (30.8%)	41 (23.4%)		
N2	41 (22.5%)	34 (19.4%)		
N3	31 (17.0%)	43 (24.6%)		
**M stage**				
M0	165 (92.2%)	165 (93.8%)	0.711	
M1	14 (7.8%)	11 (6.2%)		
**Histological type**				
Diffuse Type	31 (16.5%)	32 (17.2%)	**0.017**	exact
Mucinous Type	6 (3.2%)	13 (7.0%)		
Not Otherwise Specified	98 (52.1%)	109 (58.6%)		
Papillary Type	2 (1.1%)	3 (1.6%)		
Signet Ring Type	4 (2.1%)	7 (3.8%)		
Tubular Type	47 (25.0%)	22 (11.8%)		
**Histologic grade**				
G1	6 (3.3%)	4 (2.2%)	**0.018**	exact
G2	81 (44.0%)	56 (30.8%)		
G3	97 (52.7%)	122 (67.0%)		
**TP53 status**				
Mut	99 (52.7%)	73 (39.7%)	**0.016**	
WT	89 (47.3%)	111 (60.3%)		
**PIK3CA status**				
Mut	33 (17.6%)	26 (14.1%)	0.446	exact
WT	155 (82.4%)	158 (85.9%)		

**Table 2:** Correlations between EDNRA expression and clinicopathologic characteristics in STAD patients

Characteristics	Total (N)	Odds Ratio (OR)	P-value
T stage (T3&T4 vs. T1&T2)	367	2.25 (1.40-3.67)	<0.001
N stage (N1&N2&N3 vs. N0)	357	0.87 (0.56-1.37)	0.554
M stage (M1 vs. M0)	355	0.79 (0.34-1.78)	0.564
Pathologic stage (Stage III & Stage IV vs. Stage I & Stage II)	352	1.38 (0.91-2.11)	0.131
Histological type (Diffuse Type vs. Tubular Type)	132	2.21 (1.09-4.52)	0.028
Histologic grade (G3 vs. G1&G2)	366	1.82 (1.20-2.79)	0.005
Primary therapy outcome (CR vs. PD&SD&PR)	317	1.13 (0.69-1.87)	0.619
Residual tumor (R1&R2 vs. R0)	329	1.32 (0.63-2.81)	0.469
Tumor status (with tumor vs. Tumor free)	337	1.24 (0.80-1.94)	0.337
TP53 status (Mut vs. WT)	372	0.59 (0.39-0.89)	0.012
PIK3CA status (Mut vs. WT)	372	0.77 (0.44-1.35)	0.367

**Table 3:** Association with overall survival (OS) and clinicopathological characteristics in TCGA patients using univariate and multivariate Cox regression analysis

Characteristics	Total (N)	HR (95% CI) Univariate analysis	P-value Univariate analysis	HR (95% CI) Multivariate analysis	P-value Multivariate analysis
T stage (T3&T4 vs. T1&T2)	362	1.719 (1.131-2.612)	**0.011**	1.034 (0.537-1.993)	0.920
N stage (N1&N2&N3 vs. N0)	352	1.925 (1.264-2.931)	**0.002**	2.183 (0.971-4.910)	0.059
M stage (M1 vs. M0)	352	2.254 (1.295-3.924)	**0.004**	0.874 (0.336-2.275)	0.783
Pathologic stage (Stage III & Stage IV vs. Stage I & Stage II)	347	1.947 (1.358-2.793)	**<0.001**	0.910 (0.448-1.850)	0.795
Histological type (Diffuse Type vs. Tubular Type)	132	1.077 (0.620-1.872)	0.793		
Histologic grade (G3 vs. G1 & G2)	361	1.353 (0.957-1.914)	0.087	1.313 (0.816-2.111)	0.261
Primary therapy outcome (CR vs. PD&SD&PR)	313	0.237 (0.163-0.344)	**<0.001**	0.522 (0.305-0.896)	**0.018**
Residual tumor (R1 & R2 vs. R0)	325	3.445 (2.160-5.494)	**<0.001**	1.540 (0.777-3.055)	0.216
Age (>65 vs. ≤65)	367	1.620 (1.154-2.276)	**0.005**	1.734 (1.105-2.720)	**0.017**
Gender (Male vs. Female)	370	1.267 (0.891-1.804)	0.188		
Tumor status (with tumor vs. Tumor free)	333	5.420 (3.640-8.071)	**<0.001**	3.642 (2.051-6.467)	**<0.001**
TP53 status (Mut vs. WT)	367	0.865 (0.621-1.205)	0.392		
PIK3CA status (Mut vs. WT)	367	0.623 (0.370-1.048)	0.075	0.569 (0.311-1.040)	0.067
EDNRA (High vs. Low)	370	1.638 (1.174-2.286)	**0.004**	1.566 (1.001-2.451)	**0.049**

**Table 4:** Univariate and multivariate cox analysis of clinical parameters and EDNRA expression with OS in STAD patients from China medical university

Variables	Univariate analysis			Multivariate analysis		
	P value	Hazard Ratio	95% confidence interval	P value	Hazard Ratio	95% confidence interval
EDNRA expression	**0.04**	2.429	1.306-4.517	**0.023**	1.849	0.944-3.621
Pathological stage	**0.01**	2.331	1.452-3.742	0.108	2.474	0.820-7.462
T classification	**0.039**	1.355	1.015-1.810	0.234	0.723	0.423-1.234
N classification	0.125	2.496	1.716-3.629			
Metastasis	**0.002**	3.501	1.555-7.884	0.422	1.632	0.494-5.386
Pathologic differentiation	0.054	0.530	0.285-0.984			
Venous invasion	0.051	1.950	0.620-3.581			
lymphatic invasion	0.076	1.443	1.263-1.638			
Age	0.831	1.067	0.587-1.941			

## Abbreviations

DEG: Differentially expressed gene
EDNRA: Endothelin Receptor Type A
FDR: False discovery rate
GEO: Gene expression omnibus
GSEA: Gene Set Enrichment Analysis
IHC: Immunohistochemistry
ICI: Immune checkpoint inhibitor
NES: Normalized enrichment score
PPI: Protein-protein Interaction
STAD: Stomach Adenocarcinoma
TCGA: The Cancer Genome Atlas
TIC: Tumor-infiltrating immune cell
TIMER: Tumor immune estimation resource
